# Activation of AKT/mammalian target of rapamycin signaling in the peripheral blood of women with premature ovarian insufficiency and its correlation with *FMR1* expression

**DOI:** 10.1186/s12958-022-00919-0

**Published:** 2022-03-05

**Authors:** Julia Rehnitz, Birgitta Messmer, Ulrike Bender, Xuan Phuoc Nguyen, Ariane Germeyer, Katrin Hinderhofer, Thomas Strowitzki, Edison Capp

**Affiliations:** 1 University Women’s Hospital Heidelberg, Department of Gynecological Endocrinology and Fertility Disorders, Heidelberg, Germany; 2 Institute of Human Genetics, University Heidelberg, Laboratory of Molecular Genetics, Heidelberg, Germany; 3 Department of Obstetrics and Gynecology, Medicine School, Universidade Federal Do Rio Grande Do Sul, Porto Alegre, Brazil

**Keywords:** Fragile X mental retardation 1 gene, AKT, Mammalian target of rapamycin, S6 kinase, Tuberous sclerosis complex 2, Premature ovarian insufficiency

## Abstract

**Background:**

The protein kinase B (AKT)/mammalian target of rapamycin (mTOR) signaling pathway regulates early follicular activation and follicular pool maintenance in female germline cells. Fragile X mental retardation 1 (*FMR1*) regulates folliculogenesis and it is variably expressed in patients with Premature Ovary Insufficiency. *FMR1* expression is supposed to be linked to AKT/mTOR signaling in an ovarian response dependent manner as demonstrated in recent *in* *vitro* and *in* *vivo* studies in the female germline *in* *vitro* and *in* *vivo*.

**Methods:**

We evaluated changes in the expression of AKT/mTOR signaling pathway genes by real time PCR in the peripheral blood of 74 patients with Premature Ovarian Insufficiency and 56 fertile controls and correlated their expression with *FMR1* expression.

**Results:**

Expression of the genes *AKT1*, *TSC2, mTOR*, and *S6K* was significantly more abundant in patients with POI than in the controls. For *AKT1*, *TSC2* and *mTOR,* gene expression was not affected by *FMR1*-CGG repeat number in the 5´-untranslated region. *FMR1* and *S6K* expression levels*,* however, were significantly upregulated in patients with POI and an *FMR1* premutation. Independent of a premutation, expression of *mTOR*, *S6K*, and *TSC2* was significantly correlated with that of *FMR1* in all patients. Furthermore, when grouped according to ovarian reserve, this effect remained significant only for *mTOR* and *S6K*, with higher significance note in patients with Premature Ovarian Insufficiency than in the controls.

**Conclusions:**

In Premature ovarian insufficiency patients, activation of AKT/mTOR signaling pathway is remarkable and putatively pathognomonic. Additionally, it seems to be triggered by an *FMR1*/*mTOR*/*S6K* linkage mechanism, most relevant in premutation carriers.

## Background

Severe or complete ovarian exhaustion before the age of 40 years is referred to as premature ovarian insufficiency (POI) or premature ovarian failure syndrome [1, 2]. It is characterized by hypergonadotropic oligo- to amenorrhea (> four months) [3]. POI development is multifactorial and is caused by various pathognomonic reasons including chromosomal and genetic aberrations, autoimmune and metabolic diseases (such as galactosaemia, and polyglandular autoimmune syndromes), infections (such as mumps), and iatrogenic treatments [2]. Approximately 1% of women are affected by POI, with 4–30% having a familial element, suggesting the involvement of a genetic component [4, 5]. Several genetic factors and signaling pathways are thought to play crucial roles at different times during folliculogenesis [6]. Folliculogenesis is affected by various factors as described before and ovarian reserves can range from high, above normal, to poor and eventually exhausted at different ages. The transition from physiologically normal to pathologically abnormal reserves is hard to detect. Therefore, identification of key functional steps in ovarian aging, folliculogenesis, and its associated disorders and ovarian reserves are highly needed.

The phosphatidylinositol-3-kinase (AKT)/mammalian target of rapamycin (mTOR) signaling pathway is essential to folliculogenesis, as it maintains the primordial follicular pool. This pathway is functionally involved in primordial follicle activation, granulosa cell (GC) proliferation, oocyte-GC, inter-GC communication, and cell cycle proliferation [7, 8]. Moreover, experimental AKT activation induces follicular maturation in patients with POI [9], and *AKT1* expression in cumulus cells might serve as a marker for a positive pregnancy outcome [10].

The *FMR1* gene (encoding fragile X-mental retardation 1 protein [FMRP], OMIM: *309,550, localized at Xq27.3) is involved in folliculogenesis and associated disorders [11]. Experimental animal studies have found that *FMR1* is required for both germ stem cell maintenance and repression of primordial germ cell differentiation [12]. Moreover, this gene contains a variable CGG base triplet in its 5´-untranslated region (UTR) that is subject to premutation (PM) during expansion of 55–200 nt. Premutation alleles and so-called gray zone alleles (45–54 repeats) are associated with the development of POI in up to 13% of patients and can expand from one generation to the next. This condition is also called fragile X POI (FXPOI, OMIM #311,360) and is the most common monogenic cause of POI [13]. Elevated *FMR1* mRNA levels in PM carriers are associated with reduced FMRP levels, reflecting a negative feedback mechanism between *FMR1* and FMRP [14], which can induce several ovarian damage mechanisms [15, 16]. Additionally, distinct *FMR1* gene expression due to different lengths of CGG repeats before premutation, defined as different genotypes, affect the ovarian reserve prior to POI [17, 18]. However, large variations in leukocyte *FMR1* expression in patients with POI without PM have also been identified [19]. Furthermore, analysis of transcriptional changes in peripheral blood of *FMR1* PM carriers demonstrated CGG repeat length-dependent downregulation of genes involved in inflammation, neuronal development, apoptosis, and proliferation. One of these highly downregulated genes was *AKT1* [20].

FMRP can bind target RNAs within the RNA-interference silencing complex (RISC) [21], thereby regulating the storage, degradation, and translation of their own gene transcripts as well as other gene transcripts. Furthermore, FMRP can bind complementary 3′-UTRs in target mRNAs via their secondary structures (G-quadruplex RNA) [22]. Some of these target genes are located within the mTOR signaling pathway, including tuberous sclerosis complex 2 (*Tsc2*) and *mTOR*, as detected in ovarian studies of *Fmr*^−/−^ mice [23].

We previously identified a putative functional linkage of *FMR1*/FMRP expression with mTOR/AKT signaling under maintenance of the *FMR1*/FMRP negative feedback loop, which can be altered by specific inhibition of mTOR [24]. In addition, we recently identified significant correlations between the expression of *FMR1* and that of *AKT1*, *TSC2*, *mTOR*, and *S6K* (encoding ribosomal protein S6 kinase) in fresh GCs from women undergoing controlled ovarian stimulation for *in vitro* fertilization/intracytoplasmic sperm injection with either a normal or a poor response [25]. However, these relationships have not yet been evaluated in the peripheral blood of patients with POI and might differ according to tissue-specific expression and disease-specific alterations. Importantly, GCs in women with POI cannot be evaluated owing to ovarian exhaustion, and the detection of genes involved in AKT/mTOR signaling and putative correlations with *FMR1* in peripheral blood might offer new perspectives for women at risk of POI and associated disorders.

Accordingly, the present study aimed to determine the expression of genes involved in the AKT/mTOR signaling pathway and their putative correlations with *FMR1* in leukocytes from the peripheral blood of patients with POI and normal fertile controls (FCs), and to assess the value of these targets as predictive markers or tools in the diagnosis and prognosis of POI.

## Material and methods

### Design and patients

This prospective, observational, clinical study proceeded at the University Women’s Hospital (Heidelberg, Germany) between February 2017 and October 2020. Written, informed consent to participate in the study was obtained from 74 women with POI and 56 who were fertile (FCs). POI was defined based on the European Society for Human Reproduction and Embryology criteria [3]. The local ethics committee at Ruprecht-Karls-University, Heidelberg, Germany approved this study (ID: S-602/2013), which was conducted according to the principles of the Declaration of Helsinki (2013 amendment). Blood samples were collected from all patients and controls.

### DNA and RNA extraction

Samples of DNA and RNA were prepared in parallel from 20 mL of blood samples collected into tubes containing ethylenediaminetetraacetic acid as described previously [1]. Total RNA was primed with oligo dT using the SuperScript First-Strand Synthesis System (Invitrogen GmbH, Darmstadt, Germany; cat. no.: 11904–018) and M-MLV Reverse Transcriptase RNase H Minus, Point Mutant of Promega (Promega Corp., Madison, WI, USA; cat. no.: M 3683) to synthesize cDNA.

### Analysis of CGG repeat length

We analyzed CGG repeat lengths in the 5′-UTR of *FMR1* (NM_002024.5) exon 1 in patients with POI, using polymerase chain reaction (PCR) and an ALFexpress™ DNA sequencer (Amersham 1050; Pharmacia Biotech, Freiburg, Germany) or an ABI 3100/3130xl sequencer (Thermo Fisher Scientific Inc., Waltham, MA, USA) as described previously [17]. Since May 2020, CGG repeat lengths were analyzed using POI Triplet Repeat Primed Polymerase Chain Reaction (PCR, TP-PCR, and AmplideX® PCR/CE FMR1 Kits; Asuragen Inc., Austin, TX, USA) as per the manufacturer’s protocol, and fragments were separated using a SeqStudio Genetic Analyzer (Thermo Fisher Scientific Inc.). Electropherograms were analyzed using GeneMapper™ v. 5 software (Thermo Fisher Scientific Inc.).

### Gene expression analysis

TaqMan predesigned gene expression assays for *FMR1* (Hs00924544_m1), *AKT1* (Hs00178289_m1), *mTOR* (Hs00234508_m1), *S6K* (Hs00177357_m1), *TSC2* (Hs01020387_m1), two housekeeping genes *HPRT* and *TBP* (Hs99999909_m1; Hs00427620_m1, respectively), and TaqMan universal PCR master mix were obtained from Thermo Fisher Scientific Inc. and performed as per the manufacturer’s protocol and as described previously [25]. All samples were analyzed in triplicates under standard qPCR conditions on a Fast Forward 7500 real-time PCR system (Thermo Fisher Scientific Inc.). Relative gene expression was analyzed using the ΔΔCt method [26]. The cDNA obtained from a lymphoblastoid cell line derived from fertile women was used as the calibrator in each run.

### Statistical analysis

The data distribution was determined using Shapiro–Wilk tests. Between-group comparisons of POI vs. FCs and PM carriers vs. non-PM carriers were analyzed using Mann–Whitney tests. Not all data were normally distributed; hence, correlations were analyzed using the Spearman correlation coefficient rho (ρ).

Results are presented as medians with interquartile ranges (25^th^ to 75^th^ percentiles). All data were analyzed using the Statistical Package for the Social Sciences v. 27.0 (IBM Corp., Armonk, NY, USA), and values with *p* < 0.05 were considered statistically significant.

## Results

### Gene expression of AKT1, TSC2, mTOR, S6K, and FMR1

The expression of *FMR1* was slightly more abundant in peripheral blood from patients with POI than in FCs, although the difference was not significant. By contrast, *AKT1*, *TSC2*, *mTOR*, and *S6K* were significantly upregulated in women with POI compared with controls (Table 1).

**Table 1:** *AKT1*, *TSC2*, *mTOR*, and *S6K* expression in patients with premature ovarian insufficiency vs. fertile controls

	POI (*n* = 74)	FC (*n* = 56)	
Gene	Median (25^th^ to 75^th^ percentiles)	Median (25^th^ to 75^th^ percentiles)	*p*^a^
*FMR1*	3.539 (2.487–4.594)	3.400 (2.374–4.373)	0.382
*AKT1*	4.797 (3.972–5.553)	3.590 (1.724–5.342)	0.009
*TSC2*	3.678 (2.942–4.077)	2.917 (1.269–3.691)	0.001
*mTOR*	2.533 (1.819–3.996)	1.856 (0.517–3.255)	0.002
*S6K*	1.824 (1.564–2.281)	1.538 (0.574–2.029)	0.001

Expression of *AKT1*, *TSC2*, *mTOR*, and *S6K* (relative gene expression) in peripheral blood of patients with patients with premature ovarian insufficiency and fertile controls. Abbreviations: *POI* premature ovarian insufficiency, *FCs* fertile controls. ^a^Mann-Whitney tests

### Effects of CGG repeat length on gene expression levels in patients with POI

We found that *FMR1-*PM, *FMR1,* and *S6K* were significantly upregulated in patients with POI (Table 2).

**Table 2:** Expression of *FMR1*, *AKT1*, *TSC2*, *mTOR*, and *S6K* premutation vs non-premutation carriers

POI	POI without PM	POI with PM^b^	*P*^*a*^
*n*	67	5	
*FMR1*	3.499 (2.433–4.484)	5.376 (4.314–6.133)	0.011
*AKT1*	4.807 (4.081–5.564)	3.407 (3.305–5.401)	0.231
*TSC2*	3.730 (3.052–4.107)	2.494 (1.212–3.721)	0.141
*mTOR*	2.550 (1.878–3.917)	2.769 (1.543–4.138)	1.000
*S6K*	1.745 (1.555–2.218)	2.370 (2.291–2.909)	0.046

Relative Expression of *FMR1*, *AKT1*, *TSC2*, *mTOR*, and *S6K* in patients with premature ovarian insufficiency with and without *FMR1* premutation. Abbreviations: *PM* permutation, *POI* premature ovarian insufficiency. Data are shown as medians (25^th–^75^th^ percentiles). ^a^Mann-Whitney tests. ^b^Premutated alleles (*n*); range, 70–81 repeats

### Age

Patients were older in the FC group than in the POI group (32.5 ± 3.9 vs. 30.2 ± 7.2 y, *p* = 0.029). Therefore, the activation of mTOR/AKT signaling due to increased age in patients with POI can be excluded.

### Correlations between FMR1 and AKT1, TSC2, mTOR, and S6K

The mRNA expression of *mTOR* and *S6K* positively and significantly correlated with that of *FMR1* in all patients. These results were consistent after subgroup analysis of patients with POI and FCs. By contrast, the correlation between the expression of *TSC2* and *FMR1* was less significant, and was not observed after subgroup analysis. The expression of *AKT1* and *FMR1* did not correlate at the gene level in either group or in all patients together (Table 3).

**Table 3:** Correlations between *FMR1* and *AKT/mTOR* signaling pathway genes

All patients	Spearman correlation coefficient (*ρ*) for FMR1	*p*
*AKT*	0.136	0.122
*mTOR*	0.533	< 0.001
*S6K*	0.551	< 0.001
*TSC2*	0.217	0.013
Patients with POI		
*AKT*	0.059	0.615
*mTOR*	0.602	< 0.001
*S6K*	0.746	< 0.001
*TSC2*	0.191	0.104
FCs		
*AKT*	0.177	0.193
*mTOR*	0.388	0.003
*S6K*	0.288	0.031
*TSC2*	0.213	0.115

Correlation between *FMR1* and *AKT/mTOR* signaling pathway genes in all women, followed by subgroup analysis. Abbreviations: *POI* premature ovarian insufficiency, *FCs * fertile controls

## Discussion

This study aimed to elucidate putative changes in the AKT/mTOR signaling pathway in lymphocytes isolated from the peripheral blood of women with POI compared with women with normal fertility and to determine whether these genes correlate with *FMR1* gene expression. This is also clinically interesting as it can potentially identify new markers of ovarian reserve that can improve prediction of ovarian responses during artificial reproductive technologies. Anti-mullerian hormone and antral follicle count are currently the most relevant ovarian reserve markers, followed by age and follicle-stimulating hormone -values; however, these markers have less predictive value in cases of deviant ovarian responses, such as high or poor responses [27–29]. The results observed in the present study in patients with POI are in line with our previous data from germline cells of women with different ovarian responses during controlled ovarian stimulation [24]. Thus, identification of such novel markers that can be used in addition or alternative to existing markers in different patient groups are highly needed.

The expression of *FMR1*/FMRP is controlled through a negative feedback loop [14] and FMRP, as part of the RISC, interacts with other proteins from the Argonaute family as well as with coding and noncoding RNAs to regulate the translation of various proteins [21, 22]. In the female germline, FMRP is a major regulator of folliculogenesis, and changes in gene expression, genotype, and epigenetics (variable methylation status) are associated with disordered folliculogenesis from diminished ovarian reserve until POI [13, 18, 19, 30].

Both *TSC2* and *mTOR* are putative binding partners of *FMR1*/FMRP in *Fmr*^−/−^ mice [23]. The AKT/mTOR signaling pathway plays important roles in various cellular functions via AKT activation, TSC1/TSC2 dimerization, and mTOR activation. Finally, mTOR complex 1 phosphorylates S6K, further regulating cell-specific translation. In the female germline, AKT/mTOR signaling participates in early and late follicular maturation and GC differentiation [31–33].

The present study analyzed gene expression in lymphocytes of peripheral blood from women with normal fertility and women with POI. We found significantly more abundant *AKT1*, *TSC2*, *mTOR*, and *S6K* expression in patients with POI. Premature recruitment of oocytes therefore fit the data from experimental rodent models. Indeed, elevated mTOR and S6K levels in *Fmr*-knockout mice lead to premature oocyte recruitment [34]. In addition, we observed that *mTOR* and *S6K* was correlated with *FMR1* expression in both groups, but significantly correlated in patients with POI. These results further supported the mechanism observed in experimental animals [34] and were consistent with our data from GCs of women with poor ovarian response, in whom mTOR signaling was similarly linked to *FMR1* and particularly to *S6K* [25]. As expected from the findings of previous studies, the expression of *FMR1* was significantly more abundant in patients with POI and PM than with POI without PM [9]. Notably, *S6K* was the only gene in the AKT/mTOR pathway with a simultaneous upregulation in PM carriers. These results are consistent with findings in human neuronal cells, in which S6K is supposed to be a major FMRP-phosphorylating enzyme [35] and suggest that S6K is one of the most promising linkage partner of the AKT/mTOR pathway with *FMR1*. Noteworthy, although it was not yet significant, is the downregulation of *AKT1* that was observed in patients with POI with PM compared to those without PM. These results are consistent with previous transcriptome results from affected males carrying an FMR1 PM [20], and highlight the putative role of AKT/mTOR signaling in *FMR1* related disorders, such as FXPOI and diminished ovarian reserve (DOR).

If *FMR1/*FMRP levels are functional traits in orchestrated follicular maturation, that are linked to mTOR signaling transmitted by, or triggered through mTOR and/or S6K binding, our findings might support further studies and the development of therapeutic approaches. The easy and direct detection of these targets in peripheral blood could facilitate their application as prospective diagnostic tools for women with diminished ovarian reserve and could help estimate their individual risk for POI development.

The AKT/mTOR pathway is related to longevity, and its inhibition is thought to provide protection against age-related pathologies [36]. Therefore, our results could help identify pre-existing conditions or pathological mechanisms associated with premature ovarian aging. Alternatively, our findings might reflect a compensatory mechanism targeting the activation of the last remaining follicles in POI, which would be consistent with initial therapeutic trials of activation *in vitro*, where AKT activators induced further follicular maturation and development in women with POI [9].

Our results provide the first evidence that activation of the AKT/mTOR signaling pathway in POI is putatively linked to *FMR1* in patients with POI, diagnosed using peripheral blood probes. However, our patient cohort was small and not aged matched. Therefore, studies in larger cohort with women with distinct ovarian reserves and ages are needed to evaluate this finding in the complex context of folliculogenesis and progression to POI. The impact of CGG repeat length of this postulated relationship also needs further evaluation in longitudinal studies. Moreover, the results of both groups could be affected by age, although the average age in our cohort was below 35 years. Additionally, women in the control group were slightly older than the POI group, which contradicts age related AKT activation in POI observed in our study.

## Conclusions

*FMR1* premutation is a known risk factor for the development of POI and is recommended for clinical testing during diagnosis [3]. We believe that *FMR1/*FMRP regulates proper follicular maturation not only via its premutated CGG repeat length, but also through multiple other ways. In this context, we presumed a functional linkage between *FMR1* and the AKT/mTOR signaling pathway based on the findings of previous studies [10, 17, 23–25]. In the present study, we demonstrated upregulated AKT/mTOR signaling in patients with POI that might reflect a pathognomonic reason for POI development or represent a compensatory mechanism for POI. A linkage with *FMR1/*FMRP, presumably via modified S6K and mTOR binding, could be a functional regulator of this pathogenic mechanism and is perhaps most relevant in PM carriers, and could perhaps be more predictive than age as ovarian reserve marker in patients below 40 years of age. However, further studies are needed to elucidate the regulatory mechanisms. The detectability of these conditions in the peripheral blood of patients reflects conditions within the germline, and offers further perspectives in diagnostics and pathognomonic investigation of POI.

## Data Availability

The datasets used and analyzed during the current study are available from the corresponding author on reasonable request.

## Abbreviations

AKT/mTOR: Protein kinase B/mammalian target of rapamycin
DOR: Diminished ovarian reserve
FC: Fertile control
*FMR1*: Fragile X mental retardation 1
FMRP: Fragile X mental retardation 1 protein
FXPOI: Fragile X-associated POI
GC: Granulosa cell
POI: Premature ovary insufficiency
PM: Premutation
TSC2: Tuberous sclerosis complex 2
RISC: RNA-interference silencing complex
S6K: Ribosomal protein S6 kinase
UTR: Untranslated region
